# A breakthrough invasive *Aspergillus granulosus* infection following azole antifungal therapy in a patient with primary myelofibrosis: case report

**DOI:** 10.1016/j.mmcr.2026.100782

**Published:** 2026-03-14

**Authors:** Watchapon Jaroensawang, Piriyaporn Chongtrakool, Methee Chayakulkeeree, Anupop Jitmuang

**Affiliations:** a Division of Infectious Diseases and Tropical Medicine, Department of Medicine, Faculty of Medicine Siriraj Hospital, Mahidol University, 2 Wanglang Road, Bangkoknoi, Bangkok, Thailand; b Department of Microbiology, Faculty of Medicine Siriraj Hospital, Mahidol University, 2 Wanglang Road, Bangkoknoi, Bangkok, Thailand

**Keywords:** *Aspergillus granulosus*, Breakthrough infection, Dissemination, Invasive aspergillosis, Non-transplant individual, Myelofibrosis

## Abstract

Invasive aspergillosis (IA) primarily affects immunocompromised patients, with *Aspergillus granulosus* being a rare cause. Most cases occur in transplant recipients, and breakthrough infections may develop after azole therapy due to cryptic species and antifungal resistance. Here, we present the first non-transplant case of breakthrough *A. granulosus* infection following azole therapy with a fatal outcome. This case highlights the importance of early diagnosis, accurate species identification, and appropriate antifungal treatment for breakthrough invasive fungal infections.

## Highlights

• *Aspergillus granulosus* is a rare cause of invasive aspergillosis (IA).
• It can lead to breakthrough IA after azole therapy.
• Its cryptic phenotypes and antifungal resistance challenge the management.
• Early diagnosis with appropriate antifungal therapy is crucial to improve outcomes.

## 1 Introduction

Invasive aspergillosis (IA) is a severe fungal infection that predominantly affects immunocompromised individuals, including those with hematologic malignancies, prolonged neutropenia, organ transplants, corticosteroid or immunosuppressive therapy, advanced AIDS, or chronic granulomatous disease [1]. *Aspergillus fumigatus* is the most common pathogen in both pulmonary and extrapulmonary IA [2]. However, infections may also arise from non-*fumigatus* species such as *A. flavus*, *A. niger*, *A. terreus*, *A. nidulans*, and *A. versicolor* [3]. Rarely, cryptic species like *A. granulosus*, belonging to the *Aspergillus* section *Usti*, can cause opportunistic invasive disease [4]. This thermotolerant species has been primarily reported in post-transplant patients, causing pulmonary, skeletal, and central nervous system infections [[5], [6], [7]]. Diagnosis and management are challenging due to its cryptic phenotypes and potential antifungal resistance. Treatment of *A. granulosus* infection is problematic due to high minimum inhibitory concentrations (MICs) to azole antifungals—the standard agents for IA therapy [4]. The efficacy of amphotericin B, alone or in combination therapy, remains unclear. IA caused by *A. granulosus* is more common among organ transplant recipients, as reported by previous studies [[5], [6], [7]], although it has not been documented in patients with myelofibrosis. Patients with myelofibrosis are particularly susceptible to invasive infections. As a chronic myeloproliferative neoplasm characterized by clonal hematopoietic stem cell proliferation, bone marrow fibrosis, and progressive cytopenias, the disease predisposes individuals to innate immune dysfunction [8,9]. Additionally, treatment with JAK inhibitors like ruxolitinib suppresses JAK–STAT–mediated cytokine signaling, resulting in impaired T-cell and natural killer (NK) cell responses; neutrophil function may also be compromised. The combined effects of disease-related immune dysregulation and treatment-induced immunosuppression increase the risk of invasive opportunistic infections [10].

Here, we describe a breakthrough disseminated *A. granulosus* infection in a woman with primary myelofibrosis who previously had invasive *A. fumigatus* infection and was receiving long-term azole therapy, highlighting the need for early recognition and aggressive management of this emerging, azole-resistant pathogen.

## 2 Case presentation

**Fig. 1:**
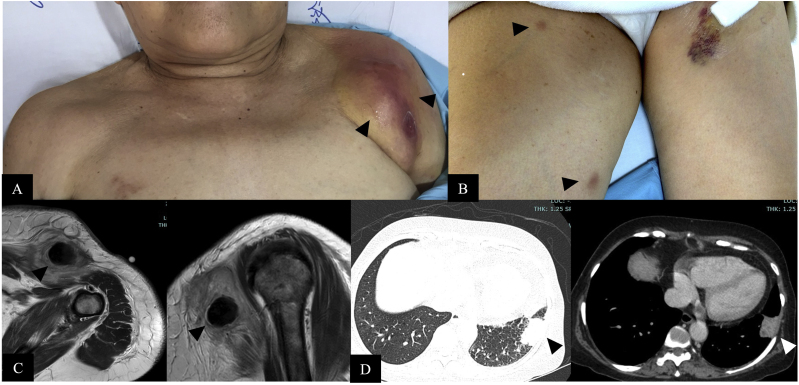
At primary diagnosis, physical examination revealed a large violaceous mass on the left shoulder (A) and painful nodules on both thighs (B). MRI showed multiple enhancing soft tissue abscesses at the left shoulder (C), and CT scan of chest revealed a heterogeneous, enhancing mass in the left lower lung (D), consistent with invasive *Aspergillus fumigatus* infection.

A 78-year-old Thai woman with primary myelofibrosis, diagnosed in February 2018 (Day 0), had been receiving intermittent blood transfusions, weekly erythropoietin, and oral ruxolitinib (30 mg/day). Seven months later (Day +210), she developed fever, nonproductive cough, and left shoulder pain persisting for three weeks. On examination, her temperature was 38.5 °C, and she had a large, violaceous, ill-defined mass on her left shoulder, along with painful subcutaneous nodules on both thighs (Fig. 1A and B). Laboratory results showed hemoglobin 8.0 g/dL, white blood cells (WBCs) 16,800/μL (80% neutrophils), and platelets 205,000/μL. Renal and liver function tests were normal. Magnetic resonance imaging (MRI) of the shoulders revealed multiple heterogeneous enhancing soft-tissue masses, suspected abscesses, ranging from 0.6 to 4.4 cm (Fig. 1C). Aspiration of the shoulder abscess and skin biopsy of the thigh nodule showed numerous hyaline septate hyphae on Gram and KOH stains. Serum galactomannan was positive (2.63). A plain chest radiography revealed left lower lung infiltrates, and a computed tomography (CT) scan demonstrated a 3 × 4 cm heterogeneous enhancing soft-tissue mass in the left lower lung (Fig. 1D). Sputum culture showed only oral flora and was negative for acid-fast organisms; however, KOH smear revealed branching septate hyphae. Based on these findings, IA was suspected. Ruxolitinib was permanently discontinued due to concerns about its potential for increased and prolonged immunosuppression, and oral voriconazole was started. All samples collected from the abscess aspirate, skin biopsy, and sputum were identified as the same fungal isolates, which showed blue-green velvety colonies and uniseriate conidial heads, consistent with *A. fumigatus*. All sample isolates were later confirmed as *A. fumigatus* using MALDI-TOF, verifying proven disseminated *A. fumigatus* infection. The patient's fever and subcutaneous lesions gradually improved with voriconazole therapy. After one month (Day +237), voriconazole was replaced with oral posaconazole due to the development of cutaneous vasculitis on both legs, likely drug-induced. Posaconazole was maintained at 800–1200 mg/day throughout the IA treatment, keeping serum levels between 1.05 and 2.68 mg/L. A follow-up chest CT at three months (Day +297) showed complete resolution of the lung mass, and all subcutaneous lesions and the shoulder abscesses had disappeared, with serum galactomannan reduced to 0.2 at approximately six months (Day +404) of antifungal therapy.

**Fig. 2:**
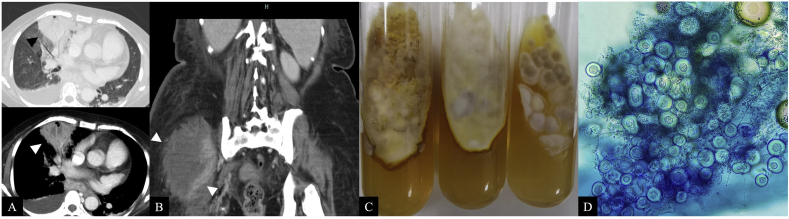
At diagnosis of breakthrough invasive *Aspergillus granulosus* infection, CT scans showed a thick-walled right lung abscess with pleural effusion (A) and a large right buttock abscess (B). Cultures from both abscess aspirate and sputum yielded brown, velvety colonies (C), and lactophenol cotton blue staining revealed numerous spherical Hülle cells without conidiation (D), confirming the disseminated fungal infection.

While continuing eight months of posaconazole treatment (Day +460), the patient developed a new fever and a persistent cough lasting three weeks, along with a large, painful, poorly defined mass in her right buttock. At that time, the hematology lab reported a WBC count of 8730/μL, with absolute neutrophils at 7410/μL and lymphocytes at 880/μL. Chest radiography revealed new infiltrates in the right lower lung. CT scans of the chest and lower abdomen showed a thick-walled abscess with pleural effusion in the right lung and a large abscess (10 × 6 × 13 cm) in the right buttock (Fig. 2A and B). Aspiration of the buttock abscess yielded 85 mL of bloody purulent fluid. Microscopic examination of the abscess aspirate and sputum samples demonstrated numerous septate hyphae, and serum galactomannan was markedly positive (4.69). Given the clinical findings, breakthrough disseminated aspergillosis was suspected. The patient was treated with a combination of oral posaconazole solution, liposomal amphotericin B (300 mg/day), and caspofungin (70 mg/day). Fungal cultures from the abscess aspirate and sputum yielded velvety, brown, cinnamon-colored colonies (Fig. 2C). Microbiological findings confirmed a proven invasive infection. Wet mounts with lactophenol cotton blue stain revealed numerous spherical Hülle cells without conidiation (Fig. 2D). Based on morphological identification, the microbiologists consider the causative fungal organism to be presumptively *A. granulosus*. To confirm speciation, the isolate was subjected to ITS1-4 PCR and sequencing, and BLAST was used to compare our fungal sequence with GenBank and CBS databases. Subsequently, the nucleotide sequencing of the isolate showed a 99-100% match to *A. granulosus* reference strains in both the GenBank and CBS databases. Therefore, molecular identification through ITS region sequencing confirmed the organism as *A. granulosus* (GenBank accession number PX446506). Antifungal susceptibility testing (Sensititre YeastOne) demonstrated extremely high MICs to all antifungal agents tested: fluconazole >256 μg/mL, 5-FC >64 μg/mL, itraconazole >16 μg/mL, voriconazole >8 μg/mL, posaconazole >8 μg/mL, amphotericin B >8 μg/mL, and echinocandins (anidulafungin, caspofungin, micafungin) all >8 μg/mL.

**Fig. 3:**
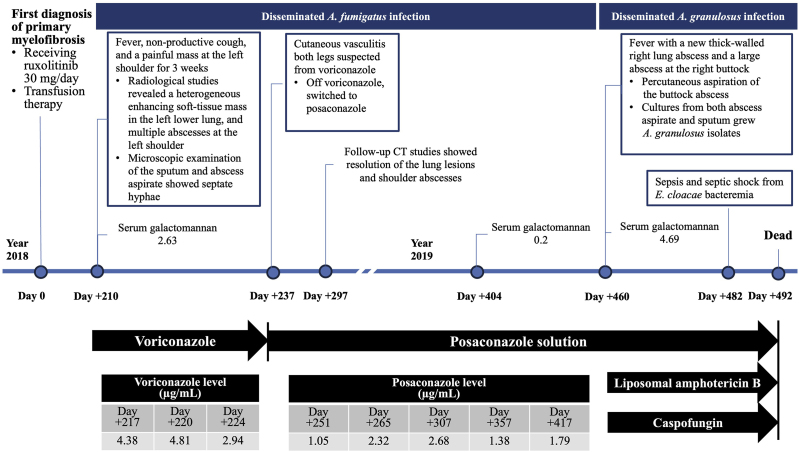
A timeline of the present case.

The combination antifungal regimen and abscess drainage provided only partial symptomatic improvement. Subsequently, the patient developed *Enterobacter cloacae* bacteremia (Day +482), treated with intravenous meropenem. Despite aggressive management, her condition worsened, and she succumbed to severe sepsis and septic shock ten days after initiating combined antifungal therapy (Day +492). A timeline outlining the clinical progression, investigations, administered antifungal agents, and monitored antifungal levels of the reported case is shown in Fig. 3.

## 3 Discussion

This case report describes a rare invasive aspergillosis caused by *A. granulosus* in a patient with severe immunosuppression due to underlying myelofibrosis and long-term ruxolitinib treatment. The case illustrates a breakthrough invasive fungal infection, as defined by the Mycoses Study Group Education and Research Consortium (MSG-ERC) and the European Confederation of Medical Mycology (ECMM) [11]. Initially infected with *A. fumigatus* and successfully treated with azoles, the patient later developed a breakthrough infection characterized by new gluteal abscesses and pulmonary lesions while on long-term posaconazole therapy, with adequate drug levels maintained for 8 months. Elevated galactomannan, fungal cultures, and the sequencing confirmed *A. granulosus*. Despite combination therapy with an azole, liposomal amphotericin B, and an echinocandin, the patient's condition did not improve and was complicated by superimposed bacterial sepsis, resulting in a fatal outcome. This case underscores the potential for azole-resistant *Aspergillus* to cause breakthrough infection during effective azole therapy, particularly in severely immunocompromised individuals.

Despite adequate posaconazole levels, this patient developed breakthrough IA, emphasizing the need to consider uncommon pathogens such as non-fumigatus *Aspergillus* species, which account for about half of breakthrough invasive fungal infections [12]. Other causative organisms include *Candida*, *Mucorales*, *Fusarium*, and rare molds or yeasts, particularly in patients with prior azole exposure [12]. Mixed fungal infections occur in 1–5% of cases [12]. Reported *Aspergillus* species causing breakthrough IA include *A. flavus*, *A. terreus*, *A. ustus* complex, *A. niger*, *A. glaucus*, *A. nidulans*, and mixed *Aspergillus* species [12]. Pulmonary involvement is most common (70–80%), while disseminated infections occur in 10–14% of cases [13]. Galactomannan antigen testing is positive in only 26–33%, culture-proven infections in 14–36% [13], and microscopic hyphae detection in merely 3.8% [12]. Because most cases (60–75%) are classified as possible or probable IA [10], diagnosis requires high clinical suspicion and multimodal evaluation. In this case, disseminated infection with a high fungal burden enabled definitive confirmation.

Breakthrough IA due to antifungal-resistant *Aspergillus* species is a major concern, particularly in patients previously treated with broad-spectrum antifungals. Limited treatment options, scarce evidence, and poor outcomes complicate management [13]. Azole resistance in *Aspergillus* occurs in 3–10% of primary IA cases, with higher rates in Europe and Asia [13]. Meanwhile, the prevalence of breakthrough infections due to resistant fungi may reach 60–80% [12]. Despite combination therapy with posaconazole, liposomal amphotericin B, and caspofungin, this patient's outcome was poor, consistent with the 35–50% mortality reported in similar cases [12]. Possible contributors include the virulence or resistance of *A. granulosus*, delayed diagnosis, and severe immunosuppression [14]. Additionally, host immune dysfunction likely played a critical role. Myelofibrosis is characterized by clonal hematopoietic stem cell proliferation, bone marrow fibrosis, and progressive cytopenias. The disease predisposes individuals to innate immune dysfunction [8,9]. The patient had primary myelofibrosis and received ruxolitinib, which inhibits the JAK/STAT pathway, thereby reducing T-cell and NK cell–mediated immunity; neutrophil function may also be impaired. This disruption of coordinated innate and adaptive antifungal immune responses may contribute to increased susceptibility to invasive infections [10,15]. Invasive fungal infections such as aspergillosis, candidiasis, and cryptococcosis have been reported in patients on ruxolitinib, usually within six months of starting therapy, with a continued cumulative risk over time [16,17]. Persistent immune dysfunction may also contribute to ongoing susceptibility [17]. After discontinuing this medication, immune function is expected to gradually recover; however, the effects of immune cell impairment may last for months after long-term treatment [18,19]. Data on the duration of sustained immunosuppression after stopping ruxolitinib are limited and vary depending on dose, treatment length, underlying disease, and co-immunosuppression. Consequently, severe immunosuppression caused by underlying myelofibrosis and long-term immune impairment from ruxolitinib are potential factors that elevate the risk of breakthrough fungal infections.

This case describes what may be the first reported instance of IA caused by *A. granulosus* in a non-transplant patient receiving ruxolitinib for primary myelofibrosis. Previously, only three cases of invasive *A. granulosus* infection have been reported, all in transplant recipients, with disease onset ranging from two weeks to six months post-transplant [[5], [6], [7]], as shown in Table 1.

**Table 1:** A summary of case reports of invasive *Aspergillus granulosus* infection.

Country, Year (Authors)	Age(yrs.)/Sex	Predisposing conditions	Clinical manifestations	Diagnosis	Treatments	MICs of antifungals (μg/mL)	Outcomes
USA, 1995 (Fakih et al.)	45/M	Post-cardiac transplant recipient	Heart failure, multiple pulmonary and skin nodules (de novo infection)	Tissue cultures and postmortem histopathology	ABD	ABD 0.29; ITRA 1.25	Died
USA, 2009 (Sutton et al.)	18/M	Post-lung transplant recipient	Multiple brain abscesses and cerebral arterial occlusions (de novo infection)	Tissue cultures and postmortem histopathology	ABLC, VORI then POSA	ABD 0.5; VORI 4; POSA 1; CAS (MEC) 0.125	Died
Germany, 2024 (Giacinta et al.)	65/M	Post-cardiac transplant recipient	Femoral osteomyelitis (de novo infection)	Tissue cultures and histopathology	ISA and Surgery	ABD 0.5; ITRA 2; VORI 2; POSA 1; ISA 1	Cure
The present case	78/F	Primary myelofibrosis with ongoing azole therapy	Multiple abscesses at lung and right buttock (breakthrough infection)	Positive serum GM Microscopic exam, tissue cultures, and histopathology	Combined LAB, POSA, CAS and drainage	ABD >8; 5-FC >64; FLU >256; ITRA >16; VORI >8; POSA >8; AND, CAS and MCF >8	Died

Abbreviations: 5-FC, flucytosine; ABD, amphotericin B deoxycholate; ABLC, amphotericin B lipid complex; AND, anidulafungin; CAS, caspofungin; F, female; FLU, fluconazole; GM, galactomannan; IA, invasive aspergillosis; ISA, isavuconazole; ITRA, itraconazole; LAB, liposomal amphotericin B; M, male; MCF, micafungin; MEC, minimal effective concentrations; MIC, minimal inhibitory concentration; POSA, posaconazole; VORI, voriconazole.

Although the mechanisms of myelofibrosis differ from those of transplantation, both conditions increase susceptibility to invasive fungal disease. Transplant patients experience iatrogenic immunosuppression, while myelofibrosis causes inherent immune dysfunction due to clonal hematopoiesis, marrow fibrosis, and cytopenias. JAK inhibition further weakens cellular immunity, potentially leading to functional deficits similar to those seen in transplant patients [19].

Most previous cases suffered from severe *A. granulosus* infection and required histopathology and culture for diagnosis [[5], [6], [7]]. However, *A. granulosus* is difficult to differentiate from other species within the *Aspergillus* section *Usti*, as they share similar colony morphology, including velvety yellow to brown-cinnamon colonies with Hülle cells [4]. Accurate identification requires PCR and sequencing of calmodulin, beta-tubulin, or ITS genes [20,21], though these tests are not routinely available. Standard susceptibility breakpoints are also lacking. According to the current case's caveat, the Sensititre YeastOne is not validated for mold antifungal susceptibility testing, and MICs obtained through this method should be interpreted with caution. Several studies show that it performs reasonably well across various antifungal agents, with important considerations depending on the drug, species, incubation time, and endpoint reading [22,23]. However, the Sensititre YeastOne is less laborious and more practical to operate in routine microbiology laboratories. Therefore, it has been applied to clinical diagnostic testing in our hospital setting, particularly for *Candida* AST. For mold isolates, it has been ordered on a case-by-case basis, particularly for isolates of special interest, which require careful interpretation and consultation with an infectious disease specialist. Species in section *Usti* exhibit variable azole susceptibility, with amphotericin B showing the most consistent activity (MIC 0.25–2 μg/mL) [21]. Terbinafine and echinocandins, particularly anidulafungin and micafungin, also demonstrate strong in vitro activity [24]. Because optimal treatment for *A. granulosus* infection is unclear, and infections often follow voriconazole or posaconazole exposure [21], early initiation of combination antifungal therapy is advisable while awaiting identification and susceptibility results. When azole-resistant aspergillosis is suspected, many experts recommend avoiding azole monotherapy and instead using liposomal amphotericin or a combination of newer azoles with echinocandins. Few clinical studies have evaluated the use of combined antifungal agents for the treatment of azole-resistant *Aspergillus*. Preclinical data suggest that the additive effect of an azole-echinocandin combination might increase susceptibility in an azole-resistant *A. fumigatus* strain, while the in vitro activity of echinocandins against *A. fumigatus* appears unaffected by the presence of azole resistance mechanisms [25]. In this case, isavuconazole and newer echinocandins were unavailable (2017–2020), so combination therapy with posaconazole, liposomal amphotericin B, and caspofungin was initiated as a salvage regimen for suspected breakthrough azole-resistant mold, pending confirmatory species identification and antifungal susceptibility testing. Despite aggressive management, the patient developed severe sepsis and died. Reported outcomes in the literature are similarly poor—two of three prior patients died, while one survived after localized femoral osteomyelitis treatment [[5], [6], [7]]. Due to the limited number of cases, prognosis and optimal therapy remain uncertain. Breakthrough *A*. *granulosus* infection in this case caused large abscesses in the right buttock and lung, indicating severe disseminated disease with a high fungal burden on microscopy. The isolate showed elevated MICs to several antifungals, making treatment more difficult. Underlying myelofibrosis and prolonged ruxolitinib treatment likely impaired host immunity and contributed to superimposed *Enterobacter cloacae* bacteremia with septic shock. Collectively, the severity of infection, high fungal burden, antifungal resistance, and impaired immunity likely complicated management and contributed to the fatal outcome.

In conclusion, *A. granulosus* is a rare pathogen that causes invasive and breakthrough infections in immunocompromised patients. Its cryptic nature and potential antifungal resistance complicate diagnosis and management, requiring early detection and combined antifungal therapy, along with restoring immune functions to prevent fatal outcomes.

## CRediT authorship contribution statement

**Watchapon Jaroensawang:** Writing – review & editing, Writing – original draft, Visualization, Data curation, Conceptualization. **Piriyaporn Chongtrakool:** Writing – review & editing, Writing – original draft, Investigation, Data curation, Conceptualization. **Methee Chayakulkeeree:** Writing – review & editing, Writing – original draft, Visualization, Supervision, Data curation, Conceptualization. **Anupop Jitmuang:** Writing – review & editing, Writing – original draft, Visualization, Validation, Supervision, Data curation, Conceptualization.

## Ethics approval and consent to participate

This case report has been exempt from requiring ethics approval by the Scientific Ethics Committee of the Siriraj Institutional Review Board (SIRB), Faculty of Medicine Siriraj Hospital, Mahidol University. Written informed consent was obtained from the patient's legal representative for the publication of this case report and any accompanying images, with confidentiality maintained. A copy of the written consent is available upon request.

## Conflict of interest

This research did not receive any specific grant from funding agencies in the public, commercial, or not-for-profit sectors.
